# Clinical efficacy of the lateral decubitus position in the treatment of irreducible trochanteric fractures with bisection of the lesser trochanter

**DOI:** 10.3389/fmedt.2026.1853499

**Published:** 2026-06-19

**Authors:** Xiang Yu, Hong-Kui Hu, Yu-Zhi Li, Xiao-Kai Li, Fan-Cheng Chen, Xu Li, Hai-Jian Lu, Bao-Qing Yu, Rong-Guang Ao

**Affiliations:** Orthopedics Department, The Seventh People’s Hospital Affiliated to Shanghai University of TCM, Shanghai, China

**Keywords:** bisection of the lesser trochanter, internal fixation, lateral decubitus position, traction table, trochanteric fracture

## Abstract

**Objective:**

To compare the clinical efficacy of the lateral decubitus position versus the supine traction table position in patients with irreducible trochanteric fractures featuring bisection of the lesser trochanter.

**Methods:**

We retrospectively analyzed 48 patients with this fracture pattern. Patients were divided into Group A (supine on traction table, *n* = 24) and Group B (lateral decubitus, *n* = 24). All underwent limited open reduction and PFNA fixation. We compared perioperative metrics (prep/time/fluoroscopy/blood loss), hospital stay, healing time, and 1-year outcomes (VAS, Harris score, reduction quality, complications).

**Results:**

Compared with the supine traction table group, the lateral decubitus group demonstrated significantly shorter preoperative preparation time (10.86 ± 2.24 vs. 18.21 ± 5.13 min, *P* < 0.001), total operative time (88.74 ± 20.72 vs. 118.45 ± 30.33 min, *P* = 0.010), fewer intraoperative fluoroscopies (58.24 ± 14.82 vs. 82.27 ± 25.44, *P* = 0.010), and reduced intraoperative blood loss (208.94 ± 67.55 vs. 288.56 ± 90.34 mL, *P* = 0.023). No significant differences were found in hospital stay, fracture healing time, or 1-year clinical/radiographic outcomes (*P* > 0.05).

**Conclusion:**

For irreducible trochanteric fractures with bisected lesser trochanters, the lateral decubitus position serves as an optimized surgical workflow and is associated with perioperative efficiency benefits over the supine traction table position, including significantly shortened operative time, reduced fluoroscopy use, and decreased blood loss.

## Introduction

Trochanteric fractures are a common type of hip fracture. Surgical treatment aims to achieve stable fracture fixation to allow for early mobilization and weight-bearing, thereby reducing complication rates and improving functional outcomes. For most of these fractures, closed reduction combined with Proximal Femoral Nail Antirotation(PFNA) fixation has become the standard treatment plan (1).

However, a subset of fractures—clinically defined as ‘irreducible'—is characterized by the failure to achieve satisfactory reduction via conventional traction or closed manipulation (2). Among these, fractures with bisection of the lesser trochanter represent a typical irreducible subtype (3). Bisection of the lesser trochanter refers to a specific two-part fracture pattern where the fracture line traverses the base of the lesser trochanter, splitting it into a proximal fragment (attached to the femoral head-neck) and a distal fragment (attached to the femoral shaft). As described by Hu et al. (3) and Zhu et al. (4), the irreducibility is biomechanically driven by soft-tissue entrapment: the iliopsoas tendon inserts on the lesser trochanter, causing the proximal fragment to rotate and lock against the superolateral femoral cortex, while the distal fragment is pulled medially by the iliacus muscle, creating a dual mechanical obstruction that resists conventional closed reduction.

For such fractures, the traditional supine position on a traction table often fails to achieve reduction due to the unique biomechanical environment of the fracture fragments and may even exacerbate displacement. Therefore, limited open reduction under direct visualization becomes necessary, and the choice of surgical position directly affects the feasibility and efficiency of the reduction procedure.

Currently, some comparative studies exist on the pros and cons of the lateral decubitus versus the supine position on a traction table for treating conventional trochanteric fractures (5). However, research specifically focused on the choice of surgical position for the “bisection of the lesser trochanter” subtype among irreducible fractures remains relatively limited. Therefore, this study aims to evaluate the technological efficacy of a standardized lateral decubitus positioning protocol as a specific procedural intervention for this mechanically complex fracture. By focusing on the methodological refinement of intraoperative positioning and fluoroscopic imaging, we seek to provide evidence-based guidance for optimizing surgical workflows, minimizing iatrogenic trauma, and enhancing the precision of intramedullary nailing in cases with lesser trochanter bisection.

## Patients and methods

### Study design

This is a retrospective study including 48 patients with irreducible trochanteric fractures with bisection of the lesser trochanter treated in our hospital from September 2021 to October 2024. Patients were allocated into two groups based on surgical position: Group A (24 patients) underwent open reduction and internal fixation on a traction table, and Group B (24 patients) underwent the procedure in the lateral decubitus position. The basic characteristics and clinical outcomes of the two groups were compared. All patients provided informed consent. All procedures in this study adhered to the ethical principles of clinical research outlined in the Declaration of Helsinki, and the study was approved by the Medical Ethics Committee of our Hospital.

### Inclusion and exclusion criteria

**Figure 1:**
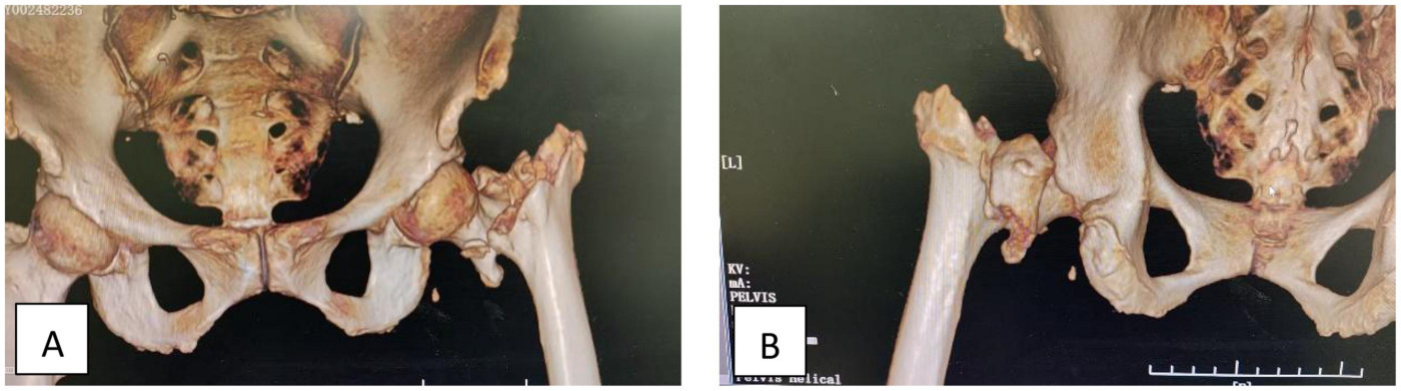
Preoperative 3D CT frontal **(A)** and dorsal **(B)** views show a simple two-part fracture of the trochanteric region without comminution (AO/OTA type 31A1.2). The fracture line extends from the greater trochanter along the trochanteric line to the medial lesser trochanter, bisecting the lesser trochanter into two parts.

The inclusion criteria were as follows: 1. Patients with a closed femoral trochanteric fracture who underwent surgery within 2 weeks after injury. 2. Patients with complete clinical data, including radiographic examinations before and after surgery. 3. Preoperative x-ray and 3D CT reconstruction images showed the trochanteric region as a simple two-part fracture without comminution (AO/OTA type 31A1.2). 4. The fracture line extended from the greater trochanter along the trochanteric line to the medial lesser trochanter, splitting the lesser trochanter into two parts (Figure 1). 5. Patients who were ambulatory and able to perform activities of daily living independently prior to the injury.

The exclusion criteria were as follows: (1) Patients with a pathologic fracture, delayed fracture, open fracture, or periprosthetic fracture. (2) Patients with multiple fractures. (3) Patients with developmental malformation of the femur. (4) Patients with femoral head necrosis with obvious collapse.

### Surgeon allocation

All procedures were performed by two independent teams of senior surgeons. Each team was led by a single attending surgeon with over 10 years of specialized experience in proximal femoral fracture management and internal fixation. One surgical team routinely performed procedures using the supine position on a traction table (Group A), while the other team specialized in the lateral decubitus position without a traction table (Group B).

### Positioning

#### Group A (traction table)

**Figure 2:**
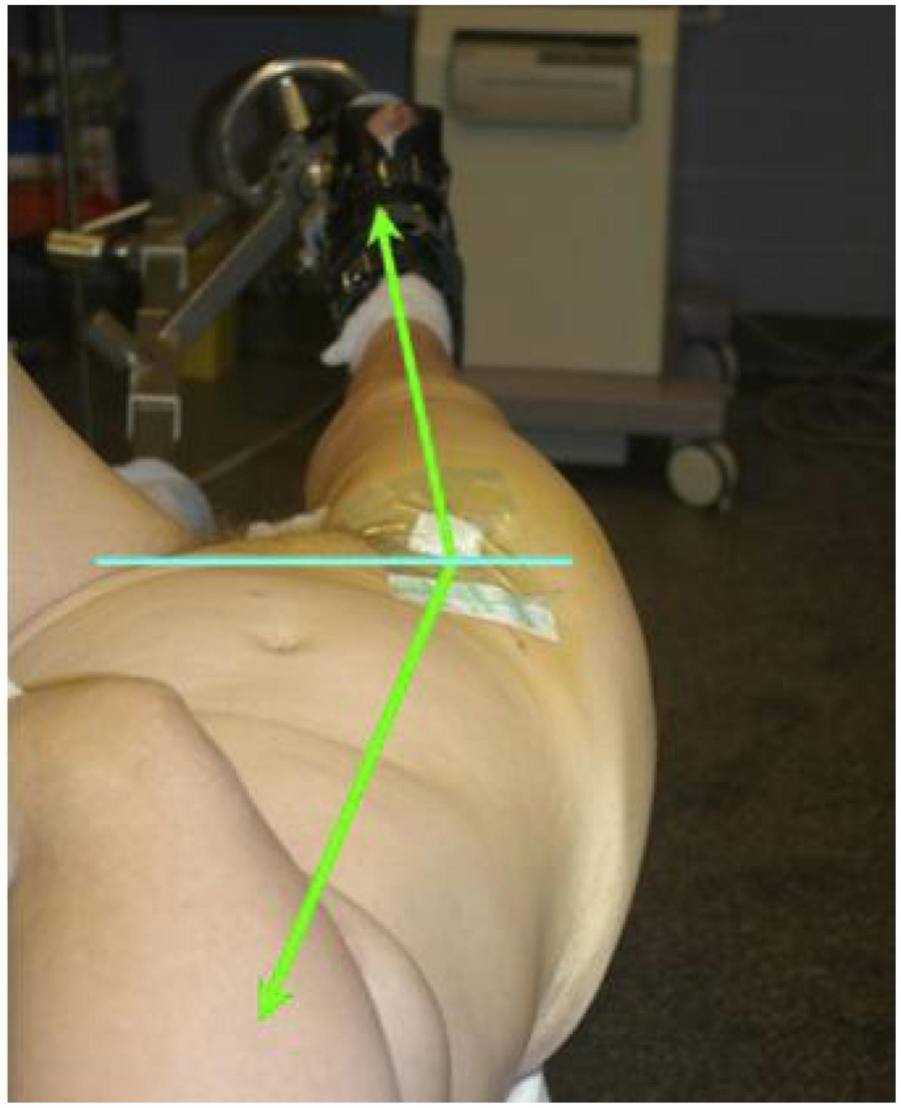
Supine traction table: upper body tilted 15°towards the healthy side (red line segment).

After general anesthesia took effect, the affected limb was secured and positioned on the traction table. The upper body was tilted 15° towards the healthy side, and the contralateral lower limb was moderately abducted (Figure 2). Following unsuccessful attempts at closed reduction under traction, traction was released, allowing the lower limb to rest in a natural, tension-free state.

#### Group B (lateral decubitus)

**Figure 3:**
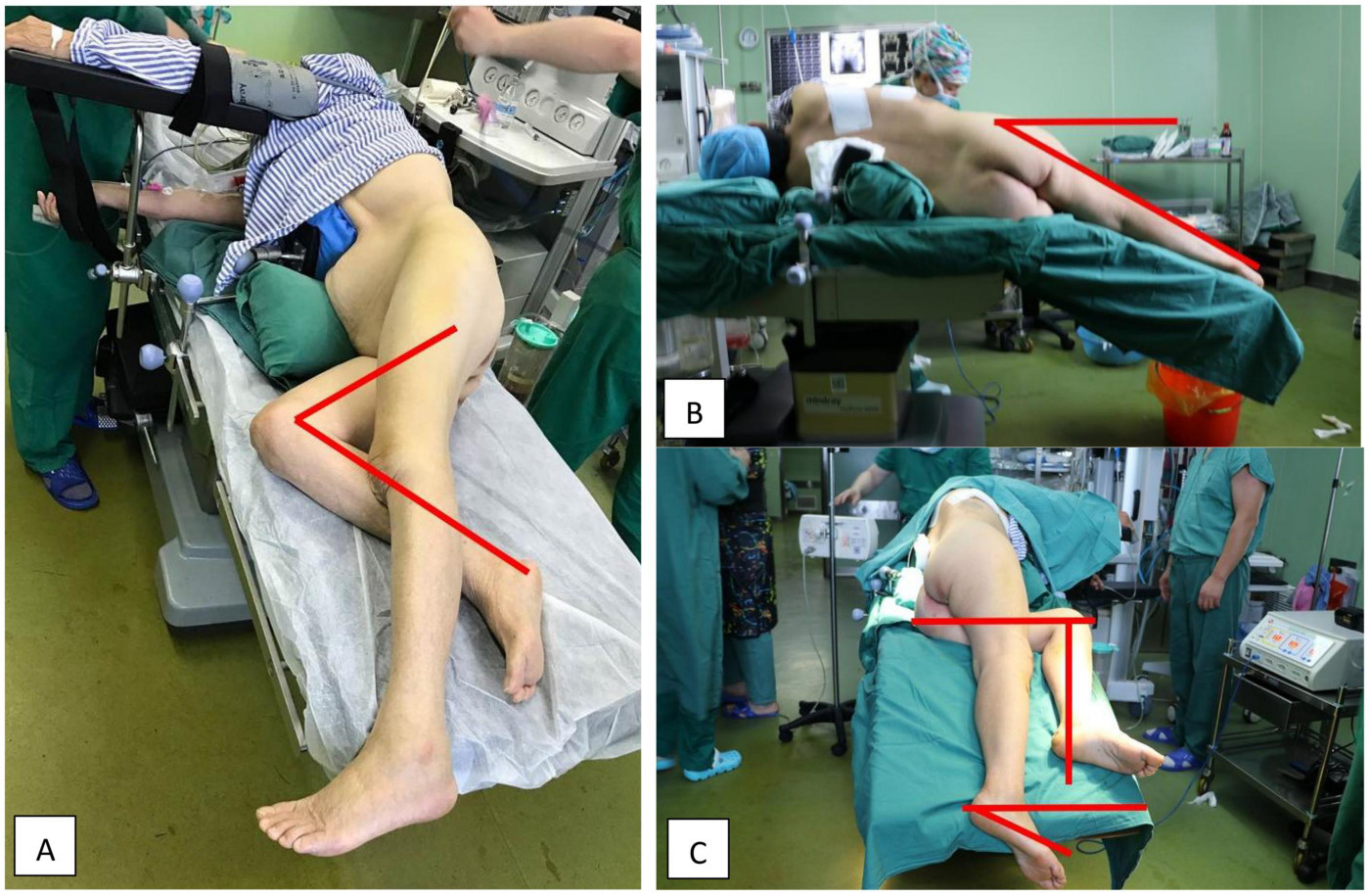
Non-traction table lateral decubitus positioning: A soft pad is placed under the chest wall on the healthy side, and the chest and back are stabilized with a fixator **(A)** the body is tilted forward 15°, the lower limb is adducted 30° **(B)** and internally rotated 15° **(C)** the contralateral lower limb is flexed at the hip and knee to 90° **(A,C)**.

**Figure 4:**
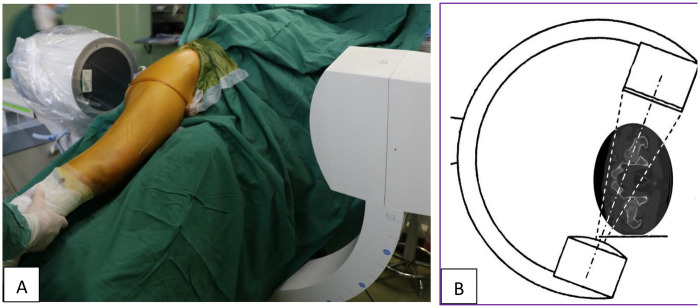
Intraoperative fluoroscopy in the lateral decubitus position: for the anteroposterior (AP) view, position the C-arm x-ray tube perpendicular to the patient's hip **(A)**; for the lateral view, tilt the C-arm 20° cephalad to obtain a coaxial image of the femoral neck and shaft **(B)**.

After general anesthesia took effect, the patient was placed in the lateral decubitus position. A soft pad was placed under the chest wall on the healthy side. The chest and back were stabilized with a fixator. The body was tilted forward 15°, the lower limb was adducted 30° and internally rotated 15°. The contralateral lower limb was flexed at the hip and knee to 90° (Figure 3). During intraoperative fluoroscopy, aligning the C-arm x-ray machine tube perpendicular to the patient's hip provided an anteroposterior view. Tilting the C-arm approximately 20° cephalad yielded a true lateral view coaxial with the femoral neck (6) (Figure 4).

### Surgical procedure

**Figure 5:**
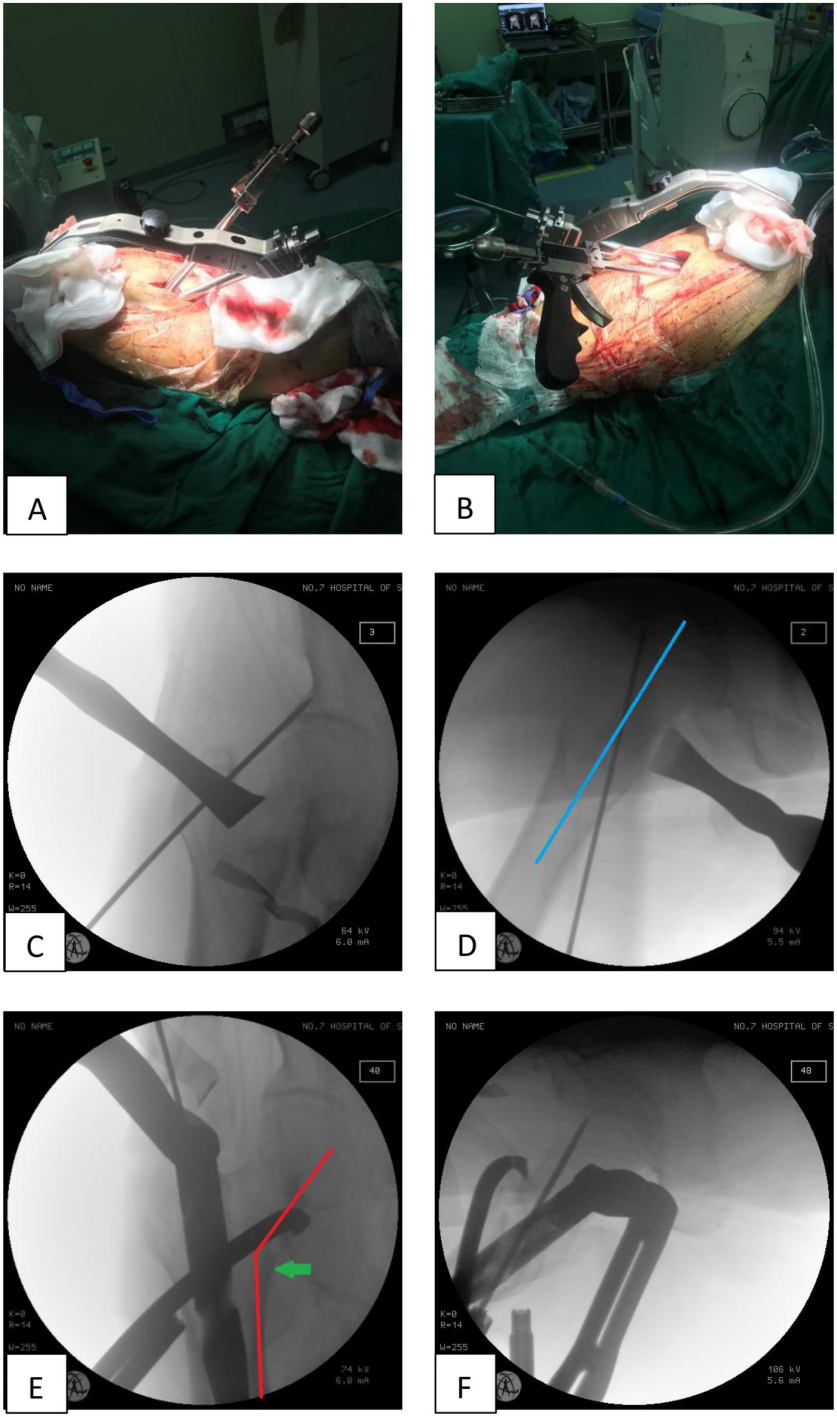
Intraoperative reduction using a pointed reduction clamp **(A,B)**; reduction assisted with a periosteal elevator under fluoroscopy **(C,D)**; reduction using a pointed reduction clamp under fluoroscopy **(E,F)**. The blue line indicates a Garden index of 180° on the lateral view. The green arrow denotes anatomic reduction of the medial cortical bone. The red line indicates a Garden index of 160° on the anteroposterior (AP) view.

The surgical procedures were similar for both groups. A 5–7 cm longitudinal skin incision was made laterally over the greater trochanter. The skin, subcutaneous tissue, and fascia lata were incised layer by layer. The vastus lateralis muscle was bluntly dissected to reach the lateral periosteum of the proximal femur. Blunt dissection was performed from the anterior surface of the trochanter to the femoral neck. The surgeon could palpate the displacement at the fracture site with a finger. Taut structures such as the iliotrochanteric ligament, anterior joint capsule, or iliacus muscle on the medial side of the femoral shaft were potential factors hindering reduction and required release to free the interlocked and entrapped soft tissues. Using tools like a periosteal elevator or a pointed reduction clamp, the fracture fragments were levered, lifted, or compressed to achieve anatomical alignment, with a Garden index measuring 160° on the anteroposterior view and 180° on the lateral view (Figure 5). Then, one or two Kirschner wires were inserted transversely or along the axis of the head-neck fragment for temporary fixation. After satisfactory fracture reduction, an entry point was made at the tip of the greater trochanter, and a guide wire was inserted into the femoral canal. Fluoroscopy confirmed the correct position of the guide wire. A PFNA nail of appropriate diameter and length was selected and inserted into the medullary canal along the guide wire. Through the aiming device, a guide wire was driven into the femoral neck. After depth measurement, the helical blade was inserted and locked. Distal locking screws were placed. Fluoroscopy confirmed maintained good fracture reduction and satisfactory implant position. The incision was thoroughly irrigated and closed in layers (fascia, subcutaneous tissue, and skin).

### Postoperative management and follow-up

**Figure 6:**
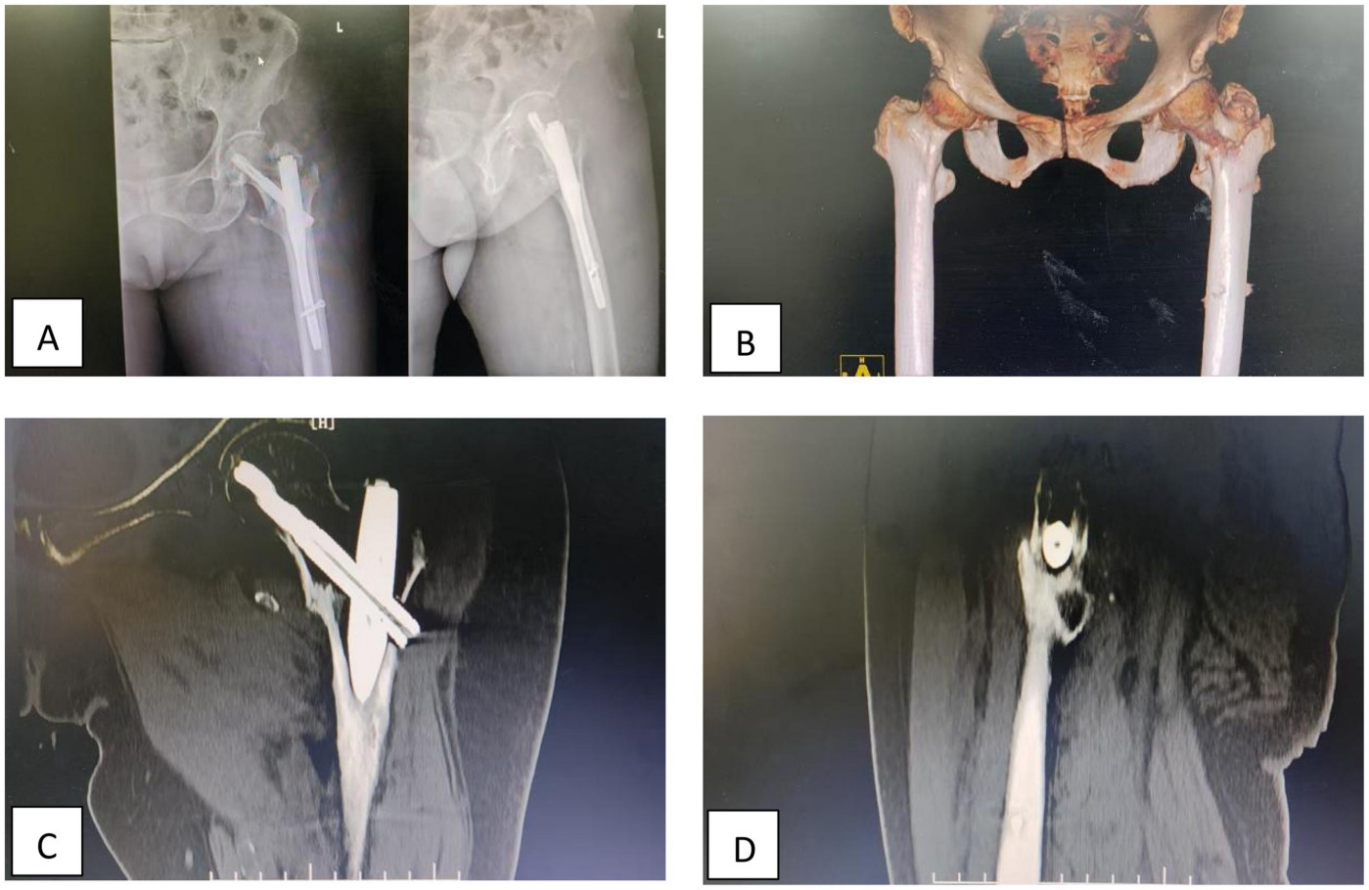
Postoperative three-day follow-up x-ray **(A)** and 3D CT **(B)** postoperative three-month anteroposterior **(C)** and lateral **(D)** CT views.

Standard postoperative anticoagulation was administered. Ankle pumps and muscle strength training were initiated within 2 weeks. Protected partial weight-bearing walking with a walker was started at 2 weeks. x-rays were reviewed at 6 weeks postoperatively, and weight-bearing was gradually increased based on callus formation. Typically, full weight-bearing was transitioned to at 3 months postoperatively after fracture healing. Regular x-rays were taken at postoperative days 3, 6 weeks, 3 months, 6 months, and 1 year to monitor fracture healing and implant status (Figure 6).

### Observation indicators

#### Baseline indicators

Age, gender, BMI, bone mineral density (BMD), fracture mechanism, cause of injury, and time from injury to surgery were collected for both groups.

#### Outcome indicators

The following were recorded for patients: preoperative preparation time, total operative time, number of intraoperative fluoroscopies, intraoperative blood loss, length of hospital stay, fracture healing time, as well as Visual Analogue Scale (VAS) score, Harris score, quality of reduction at 1-year postoperatively, and complications within 1 year.

Preoperative preparation time referred to the duration from when the anesthesia team handed over the patient to the surgical team until the skin incision was made. For Group A, this included time for patient positioning on the traction table, securing the system with appropriate traction and alignment, surgical site preparation, and sterile draping. For Group B, it included time for lateral decubitus positioning, surgical site preparation, and sterile draping.

Intraoperative blood loss was calculated by reading the total liquid volume in the suction bottle, subtracting the amount of irrigation fluid used during surgery, and adding [(total weight of used gauze and dressings (g) - pre-use dry weight (g))/1.05].

The quality of reduction was assessed using the Baumgaertner criteria (7) based on AP and lateral radiographs of the proximal femur: (1) Normal or slightly valgus neck-shaft angle on AP view, and angulation <20° on lateral view. (2) Displacement between adjacent fracture fragments <4 mm on both AP and lateral views. A “Good” reduction met both criteria. A “Fair” reduction met only one criterion. A “Poor” reduction met neither criterion. Assessments were independently evaluated by two senior radiologists in a blinded manner. To ensure objectivity, inter-observer reliability was measured using Cohen's Kappa coefficient.

Fracture healing was assessed using a combination of imaging and clinical criteria: (1) Radiographic healing was defined as the complete disappearance of the fracture line, accompanied by continuous trabecular bridging observed in serial x-rays; (2) Clinical healing was characterized by the absence of axial percussion pain and minimal pain during hip motion. Assessments were conducted by senior orthopedic physicians, while the radiographs were independently evaluated by two senior radiologists in a blinded manner. Any discrepancies in evaluations were resolved through a consensus review that integrated all available data.

### Statistical analysis

All data were managed and analyzed using IBM SPSS Statistics 26.0 software (IBM Corp., Armonk, NY, USA). The distribution of continuous variables was assessed using the Shapiro–Wilk test. Based on the distribution results, quantitative data were compared using the Mann–Whitney U Test (for non-normally distributed data) or Student's t-test (for normally distributed data), while qualitative data were analyzed using the Chi-square test. Significance levels were set at *p* < 0.05. A *post-hoc* power analysis was performed using G*Power 3.1 (Heinrich Heine University, Düsseldorf, Germany) to verify the adequacy of the sample size. Inter-observer reliability for the radiographic measurements (neck-shaft angle and fracture displacement) and the final reduction quality grading was calculated using Cohen's Kappa statistic. Kappa values were interpreted as follows: <0.20 (poor), 0.21–0.40 (fair), 0.41–0.60 (moderate), 0.61–0.80 (substantial), and 0.81–1.00 (almost perfect).

## Results

### Baseline characteristics

This study included a total of 48 eligible patients with irreducible trochanteric fractures with bisection of the lesser trochanter. They were divided into Group A (24 cases, traction table group) and Group B (24 cases, lateral decubitus group) based on surgical position. Comparison of baseline characteristics between the two groups showed no statistically significant differences in age, gender, BMI, bone mineral density, injury mechanism, injured side, or time from injury to surgery (all *P* > 0.05). This indicates that the two groups were comparable in terms of basic demographic data and fracture characteristics before surgery (Table 1).

**Table 1:** Baseline data.

Variable	Group A (Traction table)	Group B (Lateral decubitus)	*p* Value
Cases	24	24	
Age (years), Mean ± SD	54.11 ± 14.65	48.39 ± 13.20	0.326
Sex (female/male), n	16/8	14/10	0.959
BMI(kg/m2), Mean ± SD	21.61 ± 1.52	22.14 ± 1.45	0.408
BMD T-score, Mean ± SD	−1.55 ± 0.88	−1.72 ± 0.95	0.571
Mechanism of injury, *n* (%)			
Fall	8 (33.3)	8 (33.3)	0.899
Car accident	12 (50.0)	10 (41.7)	0.748
Fall from a height	4 (16.7)	6 (25.0)	0.729
Involved side (left/right), *n*	10/14	12/12	0.822
Time from fracture to surgery, Mean ± SD	1.51 ± 0.54	1.75 ± 0.74	0.429

BMI, body mass index; BMD, bone mineral density; SD, standard deviation.

Given the retrospective nature and relatively small sample size of this study, a *post-hoc* power analysis was performed to confirm the adequacy of the cohort. Based on the primary outcome of total operative time, the analysis revealed a mean of 118.45 ± 30.33 minutes in Group A versus 88.74 ± 20.72 minutes in Group B. Using an alpha level of 0.05 and a two-tailed t-test, the calculated effect size (Cohen's d) was 1.15, indicating a large clinical effect. With 24 patients in each group, the achieved statistical power (1-β) was 0.97. This result indicates that the study was sufficiently powered to detect clinically meaningful differences in perioperative efficiency between the two surgical positions.

### Outcome measurements

Comparison of perioperative and postoperative follow-up outcome indicators between the two groups showed the following results: Regarding surgical efficiency and trauma, Group B (lateral decubitus) had significantly shorter preoperative preparation time (10.86 ± 2.24 vs. 18.21 ± 5.13 min, *P* < 0.001), total operative time (88.74 ± 20.72 vs. 118.45 ± 30.33 min, *P* = 0.010), fewer intraoperative fluoroscopies (58.24 ± 14.82 vs. 82.27 ± 25.44, *P* = 0.010), and less intraoperative blood loss (208.94 ± 67.55 vs. 288.56 ± 90.34 mL, *P* = 0.023) compared to Group A (traction table), with statistically significant differences (*P* < 0.05). There were no statistically significant differences between the two groups in length of hospital stay, clinical fracture healing time, postoperative 1-year VAS pain score, or Harris hip function score (*P* > 0.05). According to the Baumgaertner criteria, both groups achieved excellent or good reduction quality, with no cases of “Poor” reduction. The Kappa coefficient was 0.89 for neck-shaft angle assessment and 0.85 for fracture displacement assessment. The overall Kappa for the final reduction quality classification (Good/Fair/Poor) was 0.87 (95% CI: 0.81–0.93). During the 1-year postoperative follow-up, all 48 patients completed scheduled assessments. Group A had 1 complication (deep vein thrombosis), and Group B had 2 complications (1 deep vein thrombosis, 1 pulmonary infection). All complications improved with symptomatic treatment. There was no statistical difference in reduction quality or complication rate between the two groups (Table 2).

**Table 2:** Comparison of outcome measures.

Outcome indicator	Group A (Traction table)	Group B (Lateral decubitus)	*p* Value	Mean difference (95% CI)
Cases	24	24		
Preparation time (min)	18.21 ± 5.13	10.86 ± 2.24	< 0.001*	−7.35 (−10.12–−4.58)
Operation time (min)	118.45 ± 30.33	88.74 ± 20.72	0.010*	−29.71 (−52.08–−7.34)
Fluoroscopies (*n*)	82.27 ± 25.44	58.24 ± 14.82	0.010*	−24.03 (−41.62–−6.44)
Blood loss (ml)	288.56 ± 90.34	208.94 ± 67.55	0.023*	−79.62 (−147.55–−10.69)
Hospital stay (days)	12.78 ± 1.56	12.44 ± 1.45	0.586	N/A
Fracture healing time (months)	3.52 ± 1.28	3.73 ± 1.40	0.705	N/A
VAS score	1.13 ± 0.63	1.21 ± 0.53	0.740	N/A
Harris score	88.45 ± 5.99	90.22 ± 6.81	0.506	N/A
Quality of fracture reduction, *n* (%)				
Good	20 (83.3)	22 (91.7)	1.000	N/A
Fair	4 (16.7)	2 (8.3)	1.000	N/A
Poor	0 (0)	0 (0)	1.000	N/A
Complications within 1 year, *n* (%)	1 (4.2)	2 (8.3)	1.000	N/A

VAS, visual analogue scale; CI, confidence interval; N/A, not applicable.

* Statistically significant difference (*P* < 0.05).

## Discussion

Irreducible trochanteric fractures, specifically those that cannot achieve satisfactory reduction through conventional traction and closed manipulation, require effective identification and classification as a foundation for developing correct surgical strategies (4). Early work by Sharma et al. (8) summarized several irreducible patterns, including fragment interlocking and bisection of the lesser trochanter, providing an initial framework for clinical identification. In recent years, with the accumulation of cases and deeper imaging analysis, the understanding of their morphology has expanded. Beyond the classic types, more complex morphologies have been included, such as femoral shaft subsidence, lateral or medial wall splitting, and comminution in the subtrochanteric region (9, 10). Among these, the specific subtype of bisection of the lesser trochanter has an incidence of approximately 2.2% to 3.6%. The irreducibility stems from the fracture line traversing the weak area of the iliopsoas tendon insertion on the lesser trochanter. This causes the proximal and distal fragments to interlock, with the proximal fragment caught on the superolateral cortex and the distal fragment entrapped medially by soft tissues under muscle traction, creating a dual mechanical obstruction that makes conventional closed reduction difficult (3). A deep understanding of the above classification and injury mechanisms allows clinicians to accurately predict the main source of reduction difficulty preoperatively. This directly guides the choice of surgical position and the planning of reduction techniques, systematically avoiding futile closed attempts and improving surgical efficiency and safety.

Multiple studies have shown that for intramedullary nailing of trochanteric fractures, the lateral decubitus position has clear advantages in shortening preoperative preparation time, total operative time, and reducing the number of intraoperative fluoroscopies. For example, the study by Sonmez et al. (11) confirmed that the lateral decubitus group was superior to the supine traction table group in these efficiency metrics, with no significant difference in reduction quality or radiographic parameters like tip-apex distance between the groups. Similarly, the case-control study by Abulsoud et al. (12) also noted that the lateral decubitus position performed better in terms of preparation time, operative time, and blood loss, without negatively affecting tip-apex distance, neck-shaft angle, or final functional scores. Our comparative results show that surgery in the lateral decubitus position offers significant advantages in surgical efficiency, with significantly shorter preoperative preparation time, total operative time, fewer intraoperative fluoroscopies, and less blood loss compared to the traditional supine position on a traction table. These findings are likely attributable to several technical advantages of the lateral decubitus position: First, it affords the surgeon a more direct and extensive lateral surgical exposure, making limited open reduction, exploration, and release of soft tissues interposed in the fracture site more convenient (13, 14). Second, this position makes the tip of the greater trochanter easier to palpate and expose, especially in obese patients, allowing for more intuitive and accurate localization of the entry point for the intramedullary nail (5, 15). Most critically, for fractures with bisection of the lesser trochanter, the lateral decubitus position allows free adduction of the affected limb. This posture can effectively relax the iliopsoas muscle attached to the lesser trochanter fragment, alleviating its traction on the fracture fragment, thereby creating favorable conditions for releasing the anterior or medial interlocking caused by muscular tension (16). These advantages collectively explain why the lateral decubitus group achieved shorter operative times and fewer fluoroscopies.

Some literature suggests (16) that the supine position on a traction table may be more conducive to obtaining standard fluoroscopic images, thereby facilitating the achievement of more ideal parameters such as the tip-apex distance. However, the practice in this study demonstrates that standardized lateral decubitus positioning and precise imaging techniques can also acquire intraoperative images that meet the requirements for internal fixation (17). Specifically, patients were precisely placed in the lateral decubitus position on the unaffected side. The torso was stabilized with positioning pads and a fixator, and the affected limb was placed at specific angles of forward tilt, adduction, and internal rotation. This posture not only helps relax the iliopsoas muscle and facilitates reduction maneuvers but, more importantly, establishes a stable geometric foundation for standardized C-arm imaging. During surgery, by adjusting the C-arm so that its tube is perpendicular to the patient's hip, a true anteroposterior image is obtained. Tilting the C-arm approximately 20° cephalad yields a clear lateral image coaxial with the femoral neck (18). This standardized operational protocol effectively overcomes potential projection angle deviations inherent to the lateral decubitus position, ensuring the accuracy and reproducibility of fluoroscopic images. Therefore, this study indicates that, regardless of the position chosen, a validated, standardized protocol for patient positioning and fluoroscopic imaging must be strictly followed. When these technical details are ensured, the lateral decubitus position can similarly provide reliable imaging guidance for accurate implant placement, achieving biomechanical fixation effects comparable to those of the supine position on a traction table.

Based on these findings, we propose that the identification of “bisection of the lesser trochanter” on preoperative 3D-CT should serve as a definitive indication to bypass the traction table. Given the inherent soft-tissue entrapment and mechanical obstruction in this specific subtype, attempting conventional closed reduction on the traction table is often futile and merely increases surgical trauma. Adopting a “direct-to-lateral” strategy with limited open reduction in the lateral decubitus position effectively relaxes iliopsoas traction and provides a direct corridor for fragment realignment. This approach can effectively shorten operative time and minimize blood loss.

While this study provides preliminary evidence supporting the lateral decubitus position, its findings are constrained by the inherent limitations of a single-center, retrospective design with a relatively small sample size and a 1-year follow-up. To overcome these constraints and validate the clinical utility of this technique, prospective, multi-center, randomized controlled trials (RCTs) are imperative. Future RCTs should enroll larger, more diverse patient populations across multiple institutions to enhance statistical power and generalizability. Furthermore, these studies should extend the follow-up period beyond one year to capture late-onset complications (e.g., implant failure, osteonecrosis) and long-term functional trajectories. Implementing rigorous randomization protocols will also be essential to eliminate selection bias stemming from surgeon preference and to definitively establish the causal relationship between surgical positioning and patient outcomes.

### Limitations

This study has several limitations. First, the retrospective, non-randomized design inherently carries the risk of selection bias.

Second, the relatively small sample size, while adequately powered to detect differences in perioperative efficiency, limits the ability to draw firm conclusions regarding secondary outcomes. Third, the lack of systematic comparison of quantitative radiographic parameters restricts the comprehensive assessment of long-term mechanical safety. Finally, the 1-year follow-up duration may be insufficient to capture late-onset complications.

## Conclusion

For irreducible trochanteric fractures with bisected lesser trochanters, the lateral decubitus position was associated with perioperative efficiency benefits over the supine traction table position, including significantly shortened operative time, reduced fluoroscopy use, and decreased blood loss. We recommend this position as the primary approach for this specific fracture morphology to avoid unnecessary traction-related delays and trauma.

## Data Availability

The raw data supporting the conclusions of this article will be made available by the authors, without undue reservation.
